# Symptomatic dry eye disease and associated factors among postgraduate students in Ethiopia

**DOI:** 10.1371/journal.pone.0272808

**Published:** 2022-08-22

**Authors:** Tarekegn Cheklie Zeleke, Nebiyat Feleke Adimassu, Abiy Maru Alemayehu, Teshager Wondale Dawud, Getasew Alemu Mersha

**Affiliations:** 1 Department of Optometry, University of Gondar, School of Medicine, Tertiary Eye Care and Training Center, Gondar, Ethiopia; 2 Department of Ophthalmology, University of Gondar, School of Medicine, Tertiary eye Care and Training Center, Gondar, Ethiopia; Universidad de Monterrey Division de Ciencias de la Salud, MEXICO

## Abstract

**Background:**

Symptomatic dry eye disease is a growing public health challenge especially among those who are visual display unit (VDU) users and other long-time near activity workers. Globally, computer user adults experience a surge in the prevalence of dry eye. Data is insufficient on the prevalence of dry eye disease among postgraduate students in Ethiopia. Therefore, the present was aimed to assess the prevalence of dry eye and its associated factors among postgraduate students at the University of Gondar, Northwest Ethiopia.

**Methods:**

A cross-sectional study was conducted on a total of 423 postgraduate students who were selected based on a simple random sampling technique. Data was collected through online symptom-based ocular surface disease index questionnaire. Binary logistic regression was used to test the association and p-value of <0.05 was considered to determine the significance of the association.

**Results:**

From the total postgraduate students, 404 completed the study with a response rate of 95.5%. The prevalence of symptomatic dry eye disease was 50.5% (95% CI, 45.1%-54.9%). Average continuous visual display unit use for 2-4hours per day[AOR = 2.57 (95% CI, 1.27–5.21)] and for> 4hours per day[AOR = 3.77 (95% CI 1.87–7.59)], duration of visual display unit use for 3–5 years [AOR = 2.24 (95% CI, 1.17–4.31)], 6–8 years [AOR = 2.46 (95% CI,1.31–4.62)] and > 8 years [AOR = 3.25 (95% CI, 1.63–6.48)], average sleeping hour < 7 hours/day within last week [AOR = 2.17 (95% CI, 1.35–3.49)] and current known allergic conjunctivitis [AOR = 5.42 (95% CI, 2.43–12.10)] were significantly associated with symptomatic dry eye disease.

**Conclusion and recommendation:**

In this study, about half of postgraduate students faced symptomatic dry eye disease. Significant association was observed between symptomatic dry eye disease and average continuous hours of visual display unit use, duration of visual display unit use in years, shortage of sleep and current known allergic conjunctivitis. It is advisable for postgraduate students to limit screen exposure hour and establish regular breaking time along their exposure. It is also advisable to have optimum sleep as possible. It is also important to explore dry eye disease on a large sample incorporating clinical tests.

## Introduction

Symptomatic dry eye disease (SDED) is a multi-factorial ocular surface condition occurs because of a disturbance in the tear film layers especially in the outer lipid layer [1]. It is manifested by frequent symptoms of dryness, ocular pain, burning, visual disturbance, eye fatigue, grittiness, photophobia, soreness, irritation & tearing [1].

Currently, SDED is a growing public health challenge especially among those who are visual display unit (VDU) users and other long-time near activity workers. Based on a number of population-based estimates worldwide the prevalence of SDED ranges from 12.3% to 62.4% in the adult population [1].

A complicity of socio-demographic, behavioral, ocular & systemic factors such as; age [1–3], sex [4], contact lens (CL) wear [5–8], VDU use [6,7,9,10], self -administered topical ophthalmic medication [8,11] shortage of sleep [8,12], systemic allergies [11,13], cataract surgery [14], migraine [13], current smoking [15,16], diabetes mellitus (DM) [15,17], arthritis [12,13], thyroid diseases [1], stress/depression, asthma [13], hypertension (HTN) [18] and anti-depressant/anti-anxiety medications plays great role in the development of SDED [12,18,19]. Although SDED rarely leads to visual loss, it frequently reduces the quality of life by interrupting crucial daily activities [15,20–22].

The economic burden of SDED is very high [4,15,23,24]. In the United States alone, the annual financial burden of dry eye is comparable with other potential ocular conditions like refractive error and cataract. The overall annual cost of dry eye for the United States healthcare system was $59.24 billion from a societal & payersperspective [24].

Using VDU and other prolonged near activities, especially in the adult population, are dramatically increasing through time [6,7,9,10,25]. Post-graduate university students are among those who have extended exposure to VDUs like a computer and other long time near visual activities. Published evidence on prevalence of symptomatic dry eye disease and its associated factors in Ethiopia is scarce. Therefore, this study is aimed to fill this gap.

## Methods and materials

### Study design and period

A web-based cross-sectional study was conducted at the University of Gondar; Northwest Ethiopia from June 21 to August 21, 2020.

### Source and study population

University of Gondar is in the historic town of Gondar, Ethiopia, 738 km from the capital, Addis Ababa. It is one of the oldest and largest higher education institutions in the country that was established in 1954 as a public health college and training center. The University has five campuses namely College of Medicine and Health Sciences, Tewodros, Fasil, Maraki and Tseda. According to the data got from the University of Gondar Registrar and Alumni Directorate Office, currently, it has 3266 students in137 postgraduate programs. The university mainly delivers the teaching process of the postgraduate program with the aid of VDU. Gondar town has a tertiary eye care hospital and private clinics which are providing comprehensive and specialty eye health care services by ophthalmologists, optometrists and ophthalmic nurses.

### Exclusion criteria

- Participants without institutional e-mails or having inactive emails were excluded.

### Sample size calculation

A computer-generated simple random sampling technique was performed to select 423 samples from 3266 postgraduate students. A computer registered sampling frame was obtained and Microsoft Excel function “= RAND ()” was used to generate simple random samples from the total postgraduate students.

The sample size calculation was done using a single population proportion formula considering prevalence of SDED from a study done in Ghana 48.1% (p = 0.481)^(11)^, maximum allowable error d 5%, and 10% non-response rate, the final computed sample size was 423.

### Ethical consideration

Ethical clearance was obtained from the Ethical Review Committee of University of Gondar, College of Medicine and Health Sciences. The tool had an informed consent form telling the participants about the aim of the study and their right to refuse participation. It had also a form seeking for voluntarism of participation before heading to the questionnaire. For confidentiality, the name of the participants was not documented and data was not disclosed. Generally, the study was conducted following the tenets of the Declaration of Helsinki.

### Operational definitions

In this study, participants who had OSDI score ≥ 13 were considered as they have symptomatic dry eye disease [26]. The use of VDU among participants was ascertained through an average continuous hour of exposure to visual display unit like a computer, television, and smart phones per day [5,7]. Participants with any diagnosed systemic allergy in the past were considered as having systemic allergy [13]. Those who reported smoking at least 100 cigarettes during their lifetime and who reported they smoke every day during data collection were designated as current smokers [15]. Sleeping hour was estimated based on an average sleeping hour per day within the last week [27,28].

If study subjects had any known depression/ anxiety which lasts for > 54 months, they were considered as having depression/anxiety [29]. Study participants who claimed using prescribed anti-depression/ anti-anxiety medications for > 18 months were classified as anti-depression/ anti-anxiety medication users [29]. Participants who used an eye drops at least once a week in the previous 3 months were grouped as self-administered topical ophthalmic medication users [8].

### Assessment of dry eye disease and risk factors

In this study, a single validated symptom based questionnaire was used to assess SDED: a standard ocular surface disease index (OSDI) questionnaire with 12 questions which is a validated and repeatable tool [26].

The questionnaire has three sections. The first evaluates the frequency of symptoms, the second evaluates the effect of symptoms on daily tasks and the third section evaluates the effect of environmental factors such as windy conditions and air conditioning. The scores on the three sections were summed up to arrive at a final OSDI score (sum of the 12 questions multiplied by 25 and divided by 12, and then rounded to next integer), which ranges from 0 to 100, with higher values showing greater symptom severity [26].

Data regarding participants’ socio demography, behavioral, ocular and systemic factors were also obtained through online questionnaire completed by study subjects. Because of the current pandemic (Covid-19), the data were collected through an online English version standard OSDI questionnaire prepared with Microsoft forms and administered through students’ institutional e-mail. The tool was pretested on 5% of the sample at Bahir Dar University and relevant amendment of the questionnaire was performed thereafter. Completeness and consistency of the collected data were ensured.

The questionnaires which were filled within a short period (< 1/3 median average time) were rejected and questions which should be filled were marked as ‘required’ that participants couldn’t skip to the next question without filling it. This tool format was restricted as one participant can’t fill greater than once.

### Data processing and analysis

The online data collected with Microsoft form was downloaded as Microsoft excel (2010) form. The data was then exported to statistical package for social science (SPSS) version 20, to be cleaned, coded and analyzed. The prevalence of symptomatic dry eye with corresponding 95% CI was determined by using the OSDI score. The descriptive analysis was summarized by frequency, percent and summary statistics. Both bi-variable and multivariable binary logistic regression analyses were done to recognize factors associated with dry eye. Variables with a p-value less than 0.2 at bi-variable analysis were considered for the multivariable analysis.

Finally, those variables with p< 0.05 at multivariable logistic regression were considered as statistically significant. The magnitude of the association was determined using an adjusted odds ratio with a 95% confidence interval.

## Results

### Socio demographic characteristics of postgraduate students

In this study, 404 post-graduate students were included with a response rate of 95.5%. The median age was 29 years, with an inter quartile range of 4. Most of the participants were male (75.2%), Orthodox Christianity followers (80.9%), and unmarried (63.1%). Half (50.2%) of the participants were first-year students and the median net monthly income was 208.75$USD with an IQR of 91.56$USD.

### Behavioral characteristics of postgraduate students

Nearly half (46%) of the participants were using visual display units for an average of > 4 continuous hours per day, whereas from all participants, nearly three fourth (72.8%) were using a hard copy for < 4 average continuous hours per day.

Only 12 (3%) participants were current smokers and 148 (36.6%) participants slept < 7 hours per day on average within last week.

### Clinical characteristics of postgraduate students

Of all 404 participants, 66 (16.3%) participants had a history of diagnosed current allergic conjunctivitis. Thirty-seven (9.2%) participants had a history of self-administered eye drop at least once a week within the last 3 months. Only 11 (2.7%) of participants had history of DM and only 11 (2.7%) had a history of known thyroid disease. Of the total 100 female participants, 17 (17%) were oral contraceptive users.

### Prevalence of symptomatic dry eye disease among postgraduate students

The prevalence of symptomatic dry eye disease among postgraduate students was found to be 50.5% (95% CI = 45.1% - 54.9).

### Related factors to symptomatic dry eye disease among postgraduate students

In bi-variable logistic regression, there was an association between SDED and average continuous hours of VDU use per day, duration of using VDUs in years, average sleeping hours per day with in the last week, using self-administered eye drop within the last 3 months, known allergic conjunctivitis, known systemic allergy, known migraine headache and history of diagnosed HTN.

Upon a multivariable logistic regression, participants with an average sleep duration of < 7 hours per day in the last week (2.01, 95% CI: 1.24–3.26), use of VDU for an average continuous hour of 2–4 (2.57,95% CI: 1.27–5.21)and > 4 (3.77, 95% CI 1.87–7.59), participants who have been using VDUs for 3–5 (2.24, 95% CI, 1.17–4.31), 6–8 (2.46, 95% CI, 1.31–4.62) and >8 (3.25, 95% CI 1.63–6.48) and participants with a history of current known allergic conjunctivitis (5.42, 95% CI 2.43–12.10) were significantly associated with symptomatic dry eye disease (Table 1 in S1 Annex).

## Discussion

The prevalence of SDED in this study was 50.5% (95% CI, 45.1–54.9) with a standard OSDI score. This result was in line with studies done among Ghanaian undergraduate students (48.1%) [11] and Mersin University Lecturers (52.8%) [9]. This consistency might be because of the similarity of exposure factors to study populations. For instance, age level and average hour exposure to VDU of this study were the same with the Ghanaian study and study among Mersin university lecturers respectively. Additionally, Mersin University study employed a similar tool with ours.

However, the present finding was lower compared to a hospital-based study on Mexican medical resident students (56%) [30]. The higher prevalence in Mexican medical resident students could be due to the difference in the exposure status of the study population to a potential risk factor. Medical residents encounter a shortage of sleep because of their extended night time duty more likely to experience SDED. Our finding was also lower compared to a population-based study among Saudi Arabian populations within the age range of 6 to 40 years with reported prevalence of SDED (62.4%) [1]. This difference could be due to the higher proportion of female study participants in Saudi Arabian study (65.4%). Females are more likely to be vulnerable to dry eye because of the hormonal changes and oral contraceptive use [31–33]. Besides, in the former study there was a higher proportion of participants with a history of smoking, contact lens use, diabetes mellitus, and arthritis which in turn increase the occurrence of SDED. Our study result was also lower than study done in Santiago (77.5%). This highest result could be due to the fact that, factors that affect SDED like being female, smoking history, thyroid disease, depression, antidepressant and oral contraceptive were higher as compared to our study. This difference could also be due the variety in the tool employed to assess dry eye; the previous study use DEQ-5 questionnaire with only 5 questions in contrast to our study’s OSDI tool with 12 questions, which may inflate the result [34].

On the other hand, the prevalence of SDED in this study was higher compared to a hospital-based study on Mexico City patients attending tertiary care center (43%) [35], a population-based study on Lebanon population (36.4%) [3] and another hospital based study on Indian patients (29.25%) [2]. The higher result of this study might be due to a difference of study populations. This study was done on participants with higher exposure status against to the previous studies which were conducted on hospital and community-based study subjects, who had relatively a lower exposure status for the important risk factors.

Moreover, our finding was also higher compared to a study on Iranian adult population which found that the prevalence of SDED was 18.3% [4]. The observed difference could be because the study on Iranian adult population had only considered severe and very severe dry eye symptoms (OSDI > 23). The utilization of high cutoff value in the OSDI made the prevalence low.

In this study, shortage of sleep (participants sleeping < 7 hours on average within the last week) have 2.01 times more likely to develop SDED as compared to those sleeping for ≥ 7 hours per day. This might be due to the fact that, sleep deprivation reduces parasympathetic tear secretion and tear hyper osmolality that could be linked to damage of surface epithelial cells and repeated ocular surface inflammation ultimately leads to dry eye [8,36].

As compared to those participants who use VDU for < 2 average continuous hours per day, those who use for 2–4 and > 4 hours have 2.4 and 3.2 higher chance of developing SDED, respectively. This was consistent with researches done on Japanese VDU users [7] Turkey lecturer [9] and Chinese medical students [10] found that, as the average time of VDU uses increase the chance of developing SDED increases. The possible reason for the association could be because, extended exposure to VDU has been linked with low blinking rate (< normal 10–15 per minute) and increased tear evaporation eventually leads to SDED [7].

Participants who have been using VDUs for 3–5 years, 6–8 years and > 8 years had 2.24, 2.46- and 3.25times increased risk of developing SDED respectively compared to participants using VDU for < 3 years. This is because using VDU for a longer period leads to reduction in blink rate which in turn leads to SDED [37].

Participants with the current known allergic conjunctivitis had 5.42 times more likely to develop SDED. This could be explained in a way that, allergic conjunctivitis is an inflammatory disorder of the conjunctiva which decrease goblet cell density & alter the lipid layer [38] and increase meibomian gland duct distortion [39] ultimately leads to the occurrence of dry eye. On top of that, medications for allergic conjunctivitis like antihistamine also has the potential to cause dry eye [12].

Studies have reported SDED has an association with increasing age [1–3]. However, in our study, we haven’t found any association. This could be probably due to the sole inclusion of young adult study population (23–47 years) in the current study. Thus, age and age-related factors of the dry eye could not be shown. Moreover this is supported by a similar study in Ghana [11].

### Limitation

As a limitation, using a single validated questionnaire instead of combined tool (OSDI and other objective assessments) might have affected the estimates of dry eye and make a comparison with the former studies difficult. Urging participants to report their past exposure to the risk factors such as, smoking cigarette, presence of anxiety and depression, use of eye drops and years of VDU use would cause a recall bias. In addition, given the wide spread of COVID 19 pandemic, we haven’t assessed the effect of wearing masks on dry eye, which some current studies conclude as an associated factor. Additionally, we believed that a larger sample size would be required to make a robust analysis and arrive at a better estimate of dry eye and associated factors among the vulnerable groups of population.

## Conclusion

In conclusion, about half of postgraduate students at University of Gondar had symptomatic dry eye disease. Average continuous VDU use hours of > 2 hours per day, > 3 years VDU use, a sleeping hour which is < 7 hours per day within last week and current known allergic conjunctivitis were the significantly associated factors.

## Supporting information

S1 Annex (DOCX)

S2 Annex (DOCX)

S1 File (SAV)

## Abbreviations

AOR: Adjusted Odds Ratio
CI: Confidence Interval
CL: Contact Lens
DM: Diabetes Mellitus
HTN: Hypertension
IQR: Interquartile Range
OSDI: Ocular Surface Disease Index
SDED: Symptomatic Dry Eye Disease
SPSS: Statistical Package for Social Science
USD: United States Dollar
VDU: Visual Display Unit
