# Supplementary material for: Symptomatic dry eye disease and associated factors among postgraduate students in Ethiopia
Source: PLoS One. 2022 Aug 22;17(8):e0272808. doi: 10.1371/journal.pone.0272808 (PMC9394807; doi:10.1371/journal.pone.0272808)
Supplement: S1 Annex — (DOCX) [file pone.0272808.s001.docx]

**Annex I**

Table 1: Binary logistic regression analysis of SDED among students at University of Gondar, Northwest Ethiopia, 2020 (n=404)

| **Variables** | **SDED** | | **Crude OR (95 % CI)** | **Adjusted OR (95 % CI)** |
| --- | --- | --- | --- | --- |
|  | **Yes (%)** | **No (%)** |  |  |
| **Sex** |  |  |  |  |
| Male | 147 (48.4) | 157 (51.6) | 1 | 1 |
| Female | 57 (57) | 43 (43) | 1.42 (0.90-2.23) | 1.36 (0.80-2.31) |
| **Average continuous VDU use hour** |  |  |  |  |
| <2 hours | 17 (26.6) | 47 (73.4) | 1 | 1 |
| 2-4 hours | 81 (51.3) | 77 (48.7) | 2.91(1.54-5.50) | 2.57 (1.27-5.21) * |
| >4 hours | 106 (58.2) | 76 (41.8) | 3.86(2.06-7.23) | 3.77 (1.87-7.59) ** |
| **Years of VDU use** |  |  |  |  |
| <3 years | 36 (33) | 73 (67.0) | 1 | 1 |
| 3-5 years | 47 (50.5) | 46 (49.5) | 2.07(1.17-3.66) | 2.24 (1.17-4.31) * |
| 6-8 years | 74 (51.7) | 52 (41.3) | 2.89(1.69-4.92) | 2.46 (1.31-4.62) * |
| >8 years | 47 (61.8) | 29 (38.2) | 3.29(1.78-6.06) | 3.25 (1.63-6.48) * |
| **Average sleeping hour within the last week** |  |  |  |  |
| <7 hours | 97 (65.5) | 51 (34.5) | 2.65 (1.74-4.03) | 2.17 (1.35-3.49) * |
| ≥7hours | 107 (41.8) | 149 (58.2) | 1 | 1 |
| **Self-used eyed drop** |  |  |  |  |
| Yes | 27 (73) | 10 (27) | 2.90 (1.36-6.16) | 1.55 (0.63-3.81) |
| No | 177 (48.2) | 190 (51.8) | 1 | 1 |
| **Known allergic conjunctivitis** |  |  |  |  |
| Yes | 57 (86.4) | 9 (13.6) | 8.23 (3.95-17.16) | 5.42 (2.43-12.10) ** |
| No | 147 (43.5) | 191 (56.5) | 1 | 1 |
| **Known HTN** |  |  |  |  |
| Yes | 15 (75) | 5 (25) | 3.10(1.10-8.69) | 1.70 (0.36-7.96) |
| No | 189 (49.2) | 195 (50.8) | 1 | 1 |
| **Known arteritis** |  |  |  |  |
| Yes | 14 (73.7) | 5 (26.3) | 2.88(0.70-10.25) | 3.25 (0.58-18.26) |
| No | 190 (49.4) | 195 (50.6) | 1 | 1 |
| **Known systemic allergy** |  |  |  |  |
| Yes | 34 (73.9) | 12 (26.1) | 3.13 (1.57-6.25) | 1.54 (0.66-3.60) |
| No | 170 (47.5) | 188 (52.5) | 1 | 1 |
| **Known migraine** |  |  |  |  |
| Yes | 40 (63.5) | 23 (36.5) | 1.88 (1.08-3.27) | 1.07 (0.55-2.06) |
| No | 164 (48.1) | 177 (51.9) | 1 |  |

OR = Odds Ratio, VDU = Visual Display Unit, * p < 0.05, ** p < 0.001
