# Supplementary material for: Symptomatic dry eye disease and associated factors among postgraduate students in Ethiopia
Source: PLoS One. 2022 Aug 22;17(8):e0272808. doi: 10.1371/journal.pone.0272808 (PMC9394807; doi:10.1371/journal.pone.0272808)
Supplement: S2 Annex — (DOCX) [file pone.0272808.s002.docx]

# Annex II: Questionnaire with consent form

Dear---------------, how are you?

My name is Tarekegn Cheklie and I am a postgraduate student at University of Gondar. I am here to collect a data about prevalence and associated factors of symptomatic dry eye disease among postgraduate students at University of Gondar. By believing factors that affect symptomatic dry eye disease like VDU use and other near activities becomes increasing on postgraduate students, this study comes with the aim of assessing the prevalence and associated factors of symptomatic dry eye disease; and finally it will give important recommendations about it from many perspectives.

To perform this research, your information is very important. Now if you are volunteer to spend a few minutes for answering this questionnaire, there is a questionnaire below with few questions related to this research. For the sake of confidentiality you will not write your name in the format and all information you will give will be kept strictly confidential. Your honest answers will contribute to exact fulfillment of this research.

If you want to know more, please contact the principal investigator (Tarekegn Cheklie) Tel: - 0931823913 /0985333771/

E-mail:- tarekegncheklie2691@gmail.com

So, are you voluntary to answer questions of this questionnaire?

Yes No

If yes, proceed to the question.

A semi-structured questionnaire to determine the prevalence and associated factors of symptomatic dry eye disease among postgraduate students at University of Gondar Collage of Medicine and Health Science, Ethiopia, 2020.

1. Socio-demographic factors (tick the alternative for closed and write on blank spaces for open questions)

| S/No | Variables | Answers |
| --- | --- | --- |
| 101 | Age in year | ______ |
| 102 | Sex | 1. Male 2. Female |
| 103 | Religion | 1. Orthodox 2. Muslim 3. Protestant 4. Catholic 5. Others______ |
| 104 | Marital status | 1. Not married 2. Married 3. Divorced 4. Widowed |
| 105 | Year of study | 1. First year 2. Second year 3. Third year 4. Fourth year |
| 106 | Net monthly income in birr | _______________ |

1. Behavioral and personal factors

| S/N | Questions | Answer | If no, skip to question |
| --- | --- | --- | --- |
| 201 | Do you currently use visual display units like computer, television, tablet & smart phone? | 1. Yes 2. No | 204 |
| 202 | If yes, for how many average continuous hours per day do you use? | __________ |  |
| 203 | How many years since you use these visual display units? | __________ |  |
| 204 | Do you currently use hard copies like books or handouts? | 1. Yes 2. No | 206 |
| 205 | If so, for how many average continuous hours you use it per day? | __________ |  |
| 206 | Do you smoke cigarette? | 1. Yes 2. No | 210 |
| 207 | If yes, how many years/ months since you start smoking? | ___________ |  |
| 208 | If yes, do you smoke currently? | 1. Yes 2. No |  |
| 209 | If yes for 206, do you smoke every day? | 1. Yes 2. No |  |
| 210 | For how many average hours do you sleep per day within this week? | ___________ |  |

1. Ocular factors

| S/N | Questions | Answer | If no, skip to question |
| --- | --- | --- | --- |
| 301 | Have you used any eye drop in your lifetime? | 1. Yes 2. No | 303 |
| 302 | If yes, have you used self-administered topical ophthalmic medication at least once a week in the previous 3 months? | 1. Yes 2. No |  |
| 303 | Do you have known allergic conjunctivitis (eye itching) now? | 1. Yes 2. No |  |

1. Systemic factor

| S/N | Questions | Answer | If no, skip to question |
| --- | --- | --- | --- |
| 401 | Do you have known diabetes mellitus? | 1. Yes 2. No |  |
| 402 | Do you have known systemic allergy like asthma, sinusitis, and dermatitis? | 1. Yes 2. No |  |
| 403 | Do you have known hypertension? | 1. Yes 2. No |  |
| 404 | Do you have known arteritis? | 1. Yes 2. No |  |
| 405 | Do you have known migraine head ache? | 1. Yes 2. No |  |
| 406 | Do you have known Stress or depression? | 1. Yes 2. No | 410 |
| 407 | If yes how long do you live with it? | __________ |  |
| 408 | If yes for 406, have you ever used anti-depressants /anti-anxiety? | 1. Yes 2. No |  |
| 409 | If yes, for how long did you use it? | __________ |  |
| 410 | Do you have known thyroid disease? | 1. Yes 2. No |  |
| 411 | Are you using oral contraceptive (for females only)? | 1. Yes 2. No |  |

500. If you have history of eye drop before, are you using artificial tear for dry eye disease now? **1. Yes 2. No** if yes, stop here.

1. Standard Ocular Surface Disease Index questionnaire

| Have you experienced any of the following *during the last week?* | All of the time | Most of the time | Half of the time | Some of the time | None of the time |
| --- | --- | --- | --- | --- | --- |
| 1. Fear of light (sensitive to light)? | 4 | 3 | 2 | 1 | 0 |
| 2. Foreign body sensation on eyes? | 4 | 3 | 2 | 1 | 0 |
| 3. Pain or grittiness on eyes? | 4 | 3 | 2 | 1 | 0 |
| 4. Blurring of vision? | 4 | 3 | 2 | 1 | 0 |
| 5. Reduced vision? | 4 | 3 | 2 | 1 | 0 |
| Have your eyes problems limited you in performing any of the following *during the last week?* | All of the time | Most of the time | Half of the time | Some of the time | None of the time |
| 6. Reading? | 4 | 3 | 2 | 1 | 0 |
| 7. Transportation (Traveling) at night? | 4 | 3 | 2 | 1 | 0 |
| 8. Using ATM or visual display units like computer or smart phones? | 4 | 3 | 2 | 1 | 0 |
| 9. Watching movies/ television? | 4 | 3 | 2 | 1 | 0 |
| Have your eyes felt uncomfortable in any of the following Situations *during the last week?* | All of the time | Most of the time | Half of the time | Some of the time | None of the time |
| 10. Windy conditions? | 4 | 3 | 2 | 1 | 0 |
| 11. Very dry (hot) conditions? | 4 | 3 | 2 | 1 | 0 |
| 12. Dusty conditions? | 4 | 3 | 2 | 1 | 0 |

Thank you for your participation!!!
